# Association of Gender, Painkiller Use, and Experienced Pain with Pain-Related Fear and Anxiety among University Students According to the Fear of Pain Questionnaire-9

**DOI:** 10.3390/ijerph18084098

**Published:** 2021-04-13

**Authors:** Paweł Piwowarczyk, Agnieszka Kaczmarska, Paweł Kutnik, Aleksandra Hap, Joanna Chajec, Urszula Myśliwiec, Mirosław Czuczwar, Michał Borys

**Affiliations:** 1 II Department of Anesthesiology and Intensive Care, Medical University of Lublin, 20-081 Lublin, Poland; 50159@student.umlub.pl (P.K.); miroslaw.czuczwar@umlub.pl (M.C.); michal.borys@umlub.pl (M.B.); 2 Student’s Scientific Association, II Department of Anesthesiology and Intensive Care, Medical University of Lublin, 20-081 Lublin, Poland; 54993@student.umlub.pl (A.K.); 54854@student.umlub.pl (A.H.); 58265@student.umlub.pl (J.C.); 55108@student.umlub.pl (U.M.)

**Keywords:** fear, pain, painkillers, gender, Fear of Pain Questionnaire

## Abstract

Anxiety and fear are determinants of acute and chronic pain. Effectively measuring fear associated with pain is critical for identifying individuals’ vulnerable to pain. This study aimed to assess fear of pain among students and evaluate factors associated with pain-related fear. We used the Fear of Pain Questionnaire-9 to measure this fear. We searched for factors associated with fear of pain: gender, size of the city where the subjects lived, subject of academic study, year of study, the greatest extent of experienced pain, frequency of painkiller use, presence of chronic or mental illness, and past hospitalization. We enrolled 717 participants. Median fear of minor pain was 5 (4–7) fear of medical pain 7 (5–9), fear of severe pain 10 (8–12), and overall fear of pain 22 (19–26). Fear of pain was associated with gender, frequency of painkiller use, and previously experienced pain intensity. We found a correlation between the greatest pain the participant can remember and fear of minor pain (r = 0.112), fear of medical pain (r = 0.116), and overall fear of pain (r = 0.133). Participants studying medicine had the lowest fear of minor pain while stomatology students had the lowest fear of medical pain. As students advanced in their studies, their fear of medical pain lowered. Addressing fear of pain according to sex of the patient, frequency of painkiller use, and greatest extent of experienced pain could ameliorate medical training and improve the quality of pain management in patients.

## 1. Introduction

Pain is a prevalent, debilitating condition that has enormous health and economic consequences [1]. The prevalence of pain in primary care settings is estimated to be 30% of patients, and approximately 116 million Americans suffer from chronic pain conditions [1,2]. Anxiety and fear have been recognized as influential moderators and determinants of the perception of both acute and chronic pain [3,4,5,6,7].

Approximately 20% of the adult European population has chronic pain. The financial cost to society is estimated at more than 200 billion euros in Europe and 150 billion dollars in the USA annually [8,9]. The prevalence of chronic pain in the UK ranges from 35.0% to 51.3%. The prevalence of moderate to severely disabling chronic pain ranges from 10.4% to 14.3%. Chronic pain prevalence increases with increasing age from 14.3% in 18–25 years old to 62% in the over 75 age group. Reported prevalence estimates in the UK were 14.2% for widespread chronic pain, 8.2% to 8.9% for chronic neuropathic pain, and 5.4% for fibromyalgia. Chronic pain was more common in female than male participants [10].

Fear is an emotional reaction to an identifiable, imminent threat, including injury [11]. Fear plays a potent role in avoidance and confrontation behavior and is strongly associated with fight or flight responses triggered by the brain’s limbic system. However, persistent fear, including fear of pain, can lead to serious adverse psychological and physiological sequelae. Patients with higher levels of fear of pain often report greater disability associated with chronic pain or experience of pain severity [12]. Moreover, patients with higher levels of fear report greater pain intensity in studies on muscle injury models [13]. Additionally, anxiety was found associated with intensified and more extended pain experiences in dental patients [14]. Interestingly, pain-related fear is considered a stronger predictor of acute pain than pain catastrophizing, which is characterized by the tendency to magnify the threat value of pain stimulus and to feel helpless [7].

Efficiently and effectively measuring fear and anxiety associated with pain in healthcare settings is critical for identifying patients vulnerable to pain. However, students from medical universities often report too few hours of education on pain management and pain’s pathomechanisms. This inadequacy results in discomfort when assessing or treating pain [15]. Moreover, doctors’ own pain experience often determines how they approach pain treatment among their patients [16]. Thus, medical, dentistry, and nursing students, as future healthcare providers, should be encouraged to use designed scales and assessment tools to assess fear of pain and increase awareness of the problem. Factors that could influence individual fear of pain, including the extent of past pain experiences, frequency of painkiller use, presence of chronic or mental illness, and past hospitalization, have not been thoroughly evaluated yet. Understanding factors associated with fear of pain among medical university students could also help prepare better training programs regarding pain assessment.

The Fear of Pain Questionnaire-9 (FPQ-9) is a shortened, validated survey based on the Fear of Pain Questionnaire-III (FPQ-III), designated to assess pain-related fear and anxiety [17]. This screening tool enables improved efficiency and allows healthcare professionals to examine more patients in a shorter time compared to the FPQ-III, which is demanded by developing healthcare systems in many countries [18,19]. 

According to the presented rationale, this study aimed to establish the extent of fear of pain among medical university students according to the Fear of Pain Questionnaire-9 and evaluate factors associated with pain-related fear and anxiety.

## 2. Materials and Methods

This study was approved by the Local Ethical Committee (approval No. KE- 0254/294/2020). The survey was conducted via the internet between January and February 2021 among students of Medical University in Lublin, Poland, mainly in Medical, Dental, and Public Health (nurses, midwives, and EMT) departments and was compliant with GDPR privacy policy. There was no randomization of the participants, and convenience sampling was used. To assess pain-related fear and anxiety, the FPQ-9 was used. The questionnaire was conducted in English, and only those who reported advanced English language skills were included in the survey. The FPQ-9 questionnaire consists of nine questions that evaluate the fear of experiencing pain in various clinical and daily-life situations (Appendix A).

Additionally, for the study, questions evaluating overall pain-related fear and anxiety were grouped to assess fear of minor pain, severe pain, and medical pain. Based on the answers from the survey, the endpoints measured in this study were

- fear of minor pain, which was calculated by summing the values of the items: 3, 5, 7 from the survey;
- fear of medical pain, which was calculated by summing the values of the items: 2, 4, 8 from the survey;
- fear of severe pain, which was calculated by summing the values of the items: 1, 6, 9 from the survey;
- overall fear of pain, which was calculated by summing values of all nine items from the survey (Appendix B).

The secondary goal of the study was to evaluate factors that could be associated with pain-related fear and anxiety: gender, size of the city where the subjects lived, subject of academic study, year of study, the greatest extent of experienced pain, frequency of painkiller use, presence of chronic or mental illness, and past hospitalization. We used the variable gender instead of sex because seven people from the surveyed population did not want to reveal their sexual identity. 

All statistical calculations were performed using STATISTICA 13.3 (StatSoft Inc., Tulsa, OK, USA). Categorical variables were presented as frequency rates and percentages. The data were tested for normal distribution using the Kolmogorov–Smirnov test; variables were presented as medians and interquartile ranges (IQR) due to a non-normal distribution. To compare data, we performed the Mann–Whitney U test, the Kruskal–Wallis test, and Spearman’s Rank-Order Correlation. A *p*-value of 0.05 or below was considered statistically significant. 

## 3. Results

We enrolled 717 participants, of whom 76.3% were female. In the study, 15.3% of participants had a history of chronic illness associated with pain, and 22.5% suffered or had a history of suffering from mental illness. The majority of the studied population (59.8%) had been hospitalized in the past. Median fear of minor pain was 5 (4–7), median fear of medical pain was 7 (5–9), median fear of severe pain was 10 (8–12), and median overall fear of pain was 22 (19–26). Participants’ detailed demographics and median answers with interquartile range for survey questions are presented in Table 1. 

Table 1 presents baseline characteristics of participants. Values are presented as number of participants in each subgroup and percentage of entire study population in the brackets, or as medians and interquartile range in the brackets. 

In this study, fear of minor pain was associated with more frequent use of painkillers. Participants studying medicine showed lower median fear in comparison to students from stomatology or other subjects (Table 2). 

Table 2 presents factors associated with fear of minor pain. Values are presented as medians and interquartile range in the brackets; *p*-value < 0.05 is considered statistically significant.

In our study, fear of medical pain was greater in females in comparison to males. Participants studying stomatology had the lowest fear of medical pain followed by medical students and students of other subjects. The final years of study were associated with a lower fear of medical pain among participants. Frequent users of painkillers showed a higher median fear of medical pain in contrast to infrequent ones (Table 3). 

Table 3 presents factors associated with fear of medical pain. Values are presented as medians and interquartile range in the brackets; *p*-value < 0.05 is considered statistically significant.

Fear of severe pain was more outlined in females than in males (Table 4).

Table 4 presents factors associated with fear of severe pain. Values are presented as medians and interquartile range in the brackets; *p*-value < 0.05 is considered statistically significant.

Overall fear of pain was more emphasized in females than in males. Past experience of high-intensity pain was associated with a higher median result of an overall fear of pain. Participants who reported an increased frequency of painkiller use showed greater overall fear of pain (Table 5).

Table 5 presents factors associated with overall fear of pain. Values are presented as medians and interquartile range in the brackets; *p*-value < 0.05 is considered statistically significant.

**Figure 1:**
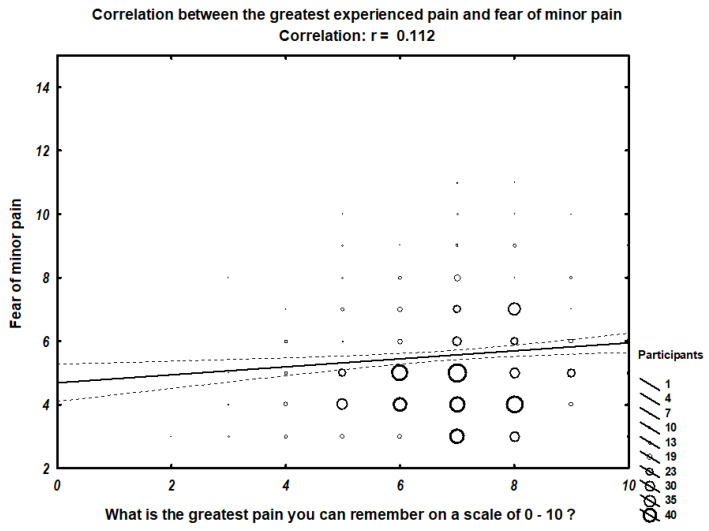
Correlation between the greatest experienced pain and fear of minor pain.

**Figure 2:**
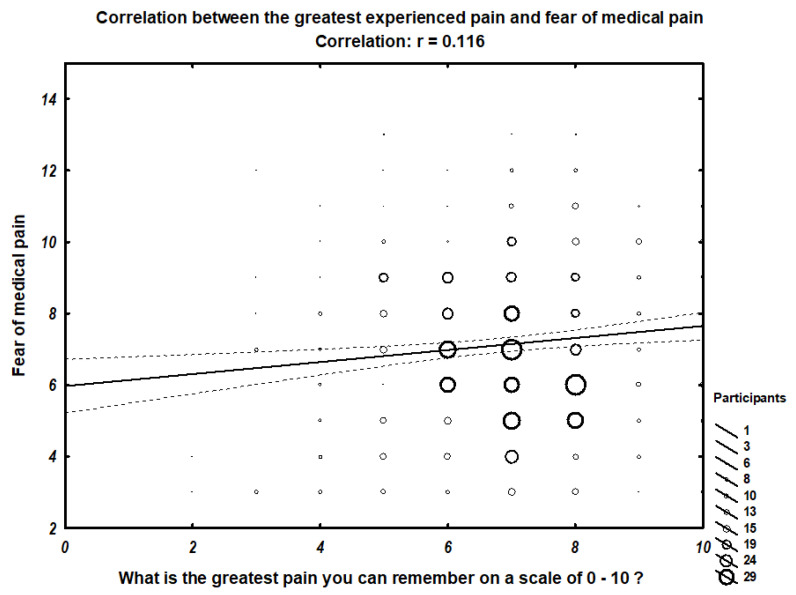
Correlation between the greatest experienced pain and fear of medical pain.

**Figure 3:**
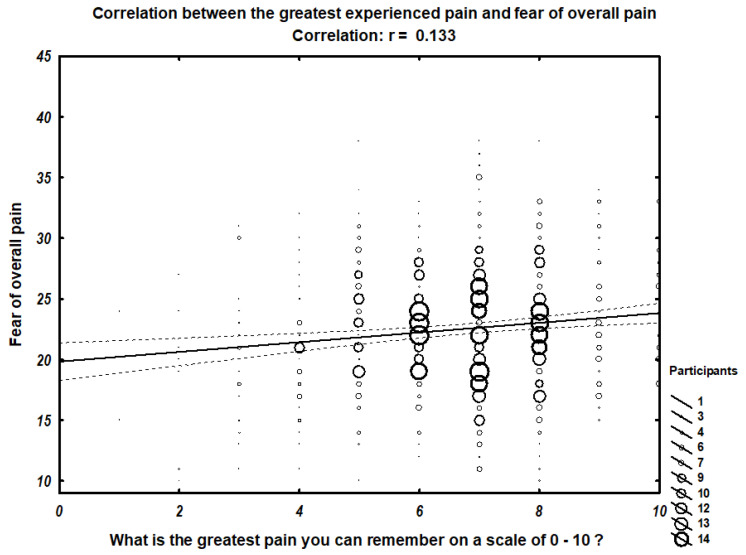
Correlation between the greatest experienced pain and fear of overall pain.

We found a correlation between the greatest pain the participant can remember and fear of minor pain (r = 0.11242) (Figure 1), fear of medical pain (r = 0.11639) (Figure 2), and overall fear of pain (r = 0.13292) (Figure 3). In contrast, no correlation with the fear of severe pain was observed. 

Figure 1 presents the scatter graph of relation between fear of minor pain and the greatest pain experienced. Size of the rings on the graph represent the number of participants within the group. 

Figure 2 presents the scatter graph of relation between fear of medical pain and the greatest pain experienced. Size of the rings on the graph represent the number of participants within the group. 

Figure 3 presents the scatter graph of relation between fear of overall pain and the greatest pain experienced. Size of the rings on the graph represent the number of participants within the group. 

The majority of our study population were medical students (57.5%), students from the nursing and midwives department (32.5%), and stomatology (10%). The fear of severe, minor, and medical pain and overall fear of pain was compared between disciplines (Table 6). 

Table 6 presents associations between type of study and type of fear of pain. Values are presented as medians and interquartile range in the brackets; *p*-value < 0.05 is considered statistically significant.

We used multivariate regression to seek an association between the results of the FPQ-9 and the greatest pain sensed by the participants. In the result, we found a model that included five quantitative variables from the FPQ-9 (answers to questions number 3, 4, 6, 7, 8) and five qualitative variables (answers to questions number 10, 11, 15, 17, 18) (Appendix A). Moreover, the sum of the FPQ-9 and each of the subscales (severe, minor, and medical pain) were also included in the model. This model’s prediction was R^2^ = 0.92, F = 449, and AIC = 2980.

## 4. Discussion 

In the presented study, we found that fear of pain was significantly associated with gender, frequent use of painkillers, and pain intensity experienced in the past. Participants studying medicine had the lowest fear of minor pain while stomatology students had the lowest fear of medical pain. As students advanced in years of studying at medical university, the median fear of medical pain decreased.

### 4.1. Association between Gender and Fear of Pain

Women and men differ in how they process pain in experimental and clinical settings [19,20,21]. However, there is still a lack of consistent evidence on psychosocial variables’ contributions, including factors that influence fear of pain, in the pain experience [22,23,24,25]. Our study results correspond with the findings presented by Robinson et al. [26], where women were more willing to report pain and consider themselves to be more sensitive to pain than males. Conversely, according to Robinson’s study, some males believed that they have higher pain endurance than women and compared to the typical male. Interestingly, after controlling for these gender role differences and anxiety, previous sex-related differences in central sensitization to pain were attenuated [27]. Robinson et al. [26] indicated that sex differences in reported pain may be attributed to underlying differences in psychosocial factors. In case of the transgender patient, the fear of discrimination/maltreatment can potentially impact fear of pain, and medical professionals should be aware of this important issue and address it appropriately [28].

### 4.2. Association between Painkiller Use and Fear of Pain

Frequent use of painkillers has been associated with increased sensitivity to pain [29]. Moreover, while the role of opioid drugs in the mechanism of opioid-induced hyperalgesia has been elucidated, in a study by Samuelson et al., reduced pain tolerance has been found for both opioid users and non-users [29,30]. Additionally, Edwards et al. [31] suggest that analgesics’ effectiveness is reduced in a state of increased pain sensitivity. Some authors postulated that increased sensitivity to pain might be a risk factor for chronic pain [32]. Moreover, according to Krebbs et al. [33], treatment with opioids is not superior to treatment with non-opioid medications for improving pain-related function over 12 months in chronic back pain or hip or knee osteoarthritis pain. Pain-related fear is a factor that may increase the risk of drug toxicity, e.g., paracetamol poisoning in dental patients [34]. On the other hand, fear of drug toxicity in cancer patients, such as in opiophobia, might prevent patients from appropriate pain management due to drug addiction’s potential risk and undesirable side effects [35,36]. The association between increased pain sensitivity, fear of pain, and frequency of painkiller use has not yet been investigated. According to our study, there is an association between fear of minor pain, fear of medical pain, and overall fear of pain and frequent use of painkillers. The causal relationship between fear of pain, sensitivity to pain, and frequency of painkiller use remains to be established. 

### 4.3. Association between the Extent of Experienced Pain and Fear of Pain

While fear of pain remains one of the leading aspects associated with pain sensitivity, multiple other factors, including attention to pain and the extent of pain experienced in the past, contribute to interindividual variance in pain processing [7,37,38,39]. Previous pain experiences, in certain circumstances, can lead to adaptation and a decrease in pain sensitivity [40,41,42,43,44,45]. On the other hand, continuous or repeated noxious stimulation can lead to sensitization to pain. Additionally, extensive past pain can alter future pain perception, and these changes may be long-lasting [46,47,48]. Interestingly, Hohmeister et al. [49] have proven that pain experience may affect sensory mechanisms and cognitive and emotional processing related to pain. Even single-episode trauma may result in altered cognitive and emotional processing of pain for years [50]. This finding might be clinically significant given that fear of pain and pain catastrophizing are known to be risk factors for the development of persistent pain [5,51,52,53]. Our study confirms an association between the greatest extent of pain experience from the past and the overall fear of pain.

### 4.4. Differences in Fear of Pain According to the Type and Year of Academic Study

In our study, both the year of study and the type of study were found to be significantly associated with fear of medical pain but not with other types of pain. The more completed years of study, and therefore the more experienced the student, the lower the fear of medical pain was. This observation could be explained by the process of obtaining more knowledge about pain management over the course of study, and consequently, more experienced students would fear medical pain less. However, in a study performed by Guivarc’h [16], dental students with more clinical experience presented less consideration for intraoperative pain. These results could imply that students undergo desensitization towards pain over their education, as they often experience a decline in empathy [48]. Pain management training should consider the potential decay of fear of medical pain among students and emphasize the importance of pain management in clinical practice. 

The influence of fear of pain on the extent of chronic pain syndromes, especially in fibromyalgia, was recently thoroughly investigated. Multiple psychological and behavioral interventions have proven their efficacy in the alleviation of chronic pain [54,55,56,57,58]. Recently, Martinez-Calderon et al. published a systematic review on the issue of intervention therapies to reduce pain-related fear in fibromyalgia [59]. 

New approaches to the management of fear of pain that involve screening of neurophysiological markers, e.g., neural oscillatory correlates of fear-related brain circuits, may be a subject of future studies. Further insight into this issue could enable the development of novel therapies that could alleviate fear and anxiety symptoms [60]. 

This study has many limitations. The baseline level of pain in response to noxious stimuli and chronic pain measures has not been evaluated in the surveyed population. The survey was performed on a non-clinical population, and caution should be taken when the presented study findings are transferred to clinical use. We used an English version of the Fear of Pain Questionnaire-9 and adhered to the self-reported English language skills of the university students who participated in the survey, as English was not their native language. Polish translation and validation of Fear of Pain Questionaire-9 should be a subject of future study. Although the survey was distributed among the Medical University students and limited to one response per person by software, the answers could be at risk of a potential violation of honesty. Other factors that could influence the participants’ fear of pain, including the participants’ ethnocultural background and economic status, were not investigated. Their impact should be elucidated in future studies. A larger sample size of the surveyed population could increase the statistical power of the associations made. 

## 5. Conclusions

While many societies and task forces studying the management of pain recommend individualized treatment approaches and fear of pain remains one of the main determinants of pain, the present study adds to how healthcare providers could tailor their treatment [61,62]. Understanding differences in fear of pain according to the patient’s sex, frequency of painkiller use, and greatest extent of experienced pain could ameliorate medical training and improve the quality of pain management in patients.

## Figures and Tables

**Table 1:** Patient baseline characteristics.

Demographics’ Features	Number of Patients (N = 717)
Gender n, (%)	
Female	547 (76.3)
Male	162 (22.6)
Prefer not to say	7 (1)
Living n, (%)	
Village	196 (27.3)
City less than 50,000 inhabitants	185 (25.7)
City less than 150,000 inhabitants	89 (12.4)
City more than 150,000 inhabitants	248 (34.6)
Type of study n, (%)	
Medicine	412 (57.5)
Stomatology	71 (10)
Public Health	234 (32.5)
Year of study n, (%)	
1	173 (24.1)
2	142 (19.8)
3	156 (21.8)
4	131 (18.3)
5	67 (9.3)
6	48 (6.7)
Greatest pain you can remember on a scale of 0–10 n, (%)	
0	2 (0.3)
1	2 (0.3)
2	8 (1.1)
3	23 (3.2)
4	41 (5.7)
5	84 (11.7)
6	117 (16.3)
7	177 (24.7)
8	146 (20.4)
9	58 (8.1)
10	58 (8.1)
Suffering from chronic illness n, (%)	
Yes	112 (15.6)
No	605 (84.4)
Mental illness present or in past n, (%)	
Yes	161 (22.5)
No	556 (77.5)
Hospitalization in the past n, (%)	
Yes	422 (58.9)
No	295 (41.1)
Use of painkillers n, (%)	
Hardly ever	301 (42)
Once a month	301 (42)
Once a week	101 (14.1)
Once a day	14 (1.9)
Fear of: Median (Interquartile range)	Study population (N = 717)
Breaking arm	3 (2–4)
Having a foot doctor remove a wart from a foot with a sharp instrument	3 (2–4)
Getting a papercut on a finger	1 (1–2)
Receiving an injection in mouth	2 (1–3)
Getting strong soap in both eyes while bathing or showering	1 (1–2)
Having someone slam a heavy car door on a hand	4 (3–4)
Gulping a hot drink before it has cooled	2 (1–3)
Receiving an injection in hip/buttocks	2 (1–3)
Falling down a flight of concrete stairs	3 (2–4)
Minor pain 3 + 5 + 7	5 (4–7)
Medical pain 2 + 4 + 8	7 (5–9)
Severe pain 1 + 6 + 9	10 (8–12)

**Table 2:** Factors associated with fear of minor pain.

Demographics’ Features	Study Population (N = 717) Median (Interquartile Range)	*p*-Value
Gender		0.87
Female	5 (4–7)	
Male	5 (4–7)	
Living		0.20
Village	5 (4–7)	
City less than 50,000 inhabitants	5 (4–7)	
City less than 150,000 inhabitants	5 (4–6)	
City more than 150,000 inhabitants	5 (4–7)	
Type of study		0.003
Medicine	5 (4–6)	
Stomatology	5 (4–7)	
Public Health	5 (4–7)	
Year of study		0.44
1	5 (4–7)	
2	5 (4–7)	
3	5 (4–7)	
4	5 (4–7)	
5	5 (4–7)	
6	5 (4–6)	
Greatest pain you can remember on a scale of 0–10		0.19
0	7 (4–10)	
1	6.5 (4–9)	
2	4 (3–6)	
3	5 (3–7)	
4	5 (4–6)	
5	5 (4–7)	
6	5 (4–6)	
7	5 (4–7)	
8	5 (4–7)	
9	5 (5–7)	
10	6 (4–8)	
Suffering from chronic illness		0.72
Yes	5 (4–7)	
No	5 (4–7)	
Mental illness present or in past		0.47
Yes	5 (4–7)	
No	5 (4–7)	
Hospitalization in the past		0.14
Yes	5 (4–7)	
No	5 (4–7)	
Use of painkillers		0.003
Hardly ever	5 (4–6)	
Once a month	5 (4–7)	
Once a week	6 (5–7)	
Once a day	6.5 (5–8)	

**Table 3:** Factors associated with fear of medical pain.

Demographics’ Features	Study Population (N = 717) Median (Interquartile Range)	*p*-Value
Gender		0.002
Female	7 (5–9)	
Male	6 (5–8)	
Living		0.25
Village	7 (5–9)	
City less than 50,000 inhabitants	7 (5–9)	
City less than 150,000 inhabitants	7 (5–8)	
City more than 150,000 inhabitants	7 (5–9)	
Type of study		0.003
Medicine	7 (5–9)	
Stomatology	7 (5–8)	
Public Health	7 (6–10)	
Year of study		0.008
1	7 (5–9)	
2	7 (6–9)	
3	7 (5–9)	
4	7 (5–9)	
5	7 (5–8)	
6	5 (4–7.5)	
Greatest pain you can remember on a scale of 0–10		0.05
0	7 (5–9)	
1	7 (6–8)	
2	4 (3–5.5)	
3	7 (3–8)	
4	6 (4–8)	
5	7.5 (5–9)	
6	7 (6–8)	
7	7 (5–9)	
8	7 (5–9)	
9	7 (5–10)	
10	7.5 (5–10)	
Suffering from chronic illness		0.77
Yes	7 (5–9)	
No	7 (5–9)	
Mental illness present or in past		0.24
Yes	7 (5–9)	
No	7 (5–9)	
Hospitalization in the past		0.66
Yes	7 (5–9)	
No	7 (5–9)	
Use of painkillers		0.004
Hardly ever	6 (5–8)	
Once a month	7 (5–9)	
Once a week	7 (5–9)	
Once a day	8.5 (6–10)	

**Table 4:** Factors associated with fear of severe pain.

Demographics’ Features	Study Population (N = 717) Median (Interquartile Range)	*p*-Value
Gender		0.0007
Female	10 (8–12)	
Male	10 (8–11)	
Living		0.92
Village	10 (8–12)	
City less than 50,000 inhabitants	10 (8–12)	
City less than 150,000 inhabitants	10 (8–12)	
City more than 150,000 inhibitants	10 (8–12)	
Type of study		0.28
Medicine	10 (8–12)	
Stomatology	10 (9–12)	
Public Health	10 (8–12)	
Year of study		0.61
1	10 (8–12)	
2	10 (8–12)	
3	10 (8–11.5)	
4	10 (8–12)	
5	10 (8–12)	
6	9 (8–11.5)	
Greatest pain you can remember on a scale of 0–10		0.07
0	8 (6–10)	
1	6 (5–7)	
2	9.5 (5–12)	
3	9 (7–10)	
4	9 (8–11)	
5	10 (8–12)	
6	11 (9–12)	
7	10 (8–12)	
8	10 (8–12)	
9	10 (8–11)	
10	10.5 (9–12)	
Suffering from chronic illness		0.35
Yes	10 (8–12)	
No	10 (8–12)	
Mental illness present or in past		0.39
Yes	10 (8–12)	
No	10 (8–12)	
Hospitalization in the past		0.66
Yes	10 (8–12)	
No	10 (8–12)	
Use of painkillers		0.17
Hardly ever	10 (8–12)	
Once a month	10 (8–12)	
Once a week	10 (9–12)	
Once a day	11.5 (9–12)	

**Table 5:** Factors associated with overall fear of pain.

Demographics’ Features	Study Population (N = 717) Median (Interquartile Range)	*p*-Value
Gender		0.003
Female	23 (19–27)	
Male	21 (17–25)	
Living		0.20
Village	23 (19.5–27)	
City less than 50,000 inhabitants	23 (18–26)	
City less than 150,000 inhabitants	21 (18–25)	
City more than 150,000 inhabitants	22 (18–26)	
Type of study		0.06
Medicine	22 (18–26)	
Stomatology	22 (20–25)	
Public Health	23 (19–27)	
Year of study		0.07
1	23 (19–27)	
2	23 (19–27)	
3	22 (18–25.5)	
4	23 (19–27)	
5	23 (18–26)	
6	21 (17–24)	
Greatest pain you can remember on a scale of 0–10		0.04
0	22 (19–25)	
1	19.5 (15–24)	
2	19.5 (11–22.5)	
3	21 (15–24)	
4	21 (17–23)	
5	23 (19–27)	
6	23 (20–25)	
7	22 (18–26)	
8	23 (19–26)	
9	22.5 (19–26)	
10	24.5 (20–29)	
Suffering from chronic illness		0.62
Yes	23 (19–26)	
No	22 (19–26)	
Mental illness present or in past		0.66
Yes	22 (19–26)	
No	22 (19–26)	
Hospitalization in the past		0.73
Yes	22 (19–26)	
No	23 (18–27)	
Use of painkillers		0.002
Hardly ever	22 (18–25)	
Once a month	23 (19–26)	
Once a week	24 (20–28)	
Once a day	26.5 (20–31)	

**Table 6:** Fear of pain between disciplines.

Type of Study	Fear of Minor Pain Median (Interquartile Range)	*p*-Value	Fear of Severe Pain Median (Interquartile Range)	*p*-Value	Fear of Medical Pain Median (Interquartile Range)	*p*-Value	Overall Fear of Pain Median (Interquartile Range)	*p*-Value
Medicine	5 (4–6)	0.003	10 (8–12)	0.28	7 (5–9)	0.003	22 (18–26)	0.06
Stomatology	5 (4–7)		10 (9–12)		7 (5–8)		22 (20–25)	
Public Health	5 (4–7)		10 (8–12)		7 (6–10)		23 (19–27)	

## Data Availability

The data presented in this study are available on request from the corresponding author. The data are not publicly available due to University’s data protection regulations.

## Appendix A. FEAR of PAIN Questionnaire-9

The items listed below describe painful experiences. Please look at each item and think about how FEARFUL you are of experiencing the PAIN associated with each item. If you have never experienced the PAIN of a particular item, please answer on the basis of how FEARFUL you expect you would be if you had such an experience. Circle one number for each item below to rate your FEAR OF PAIN in relations to each event. (1—Not at all; 2—A little; 3—A fair amount; 4—Very much; 5—Extreme).

**I FEAR the PAIN Associated with:**	**Not at All**	**A Little**	**A Fair Amount**	**Very Much**	**Extreme**
1. Breaking your arm	1	2	3	4	5
2. Having a foot doctor remove a wart from your foot with a sharp instrument	1	2	3	4	5
3. Getting a papercut on your finger	1	2	3	4	5
4. Receiving an injection in your mouth	1	2	3	4	5
5. Getting strong soap in both your eyes while bathing or showering	1	2	3	4	5
6. Having someone slam a heavy car door on your hand	1	2	3	4	5
7. Gulping a hot drink before it has cooled	1	2	3	4	5
8. Receiving an injection in your hip/buttocks	1	2	3	4	5
9. Falling down a flight of concrete stairs	1	2	3	4	5

## Appendix B

Scoring instructions for Fear of Pain Questionnaire-9:

(1) Score the Fear of Severe Pain subscale by summing values for the following items: 1, 6, 9
(2) Score the Fear of Minor Pain subscale by summing values for the following items: 3, 5, 7
(3) Score the Fear of Medical/Dental Pain subscale by summing values for the following items: 2, 4, 8
(4) Calculate the Total Score by summing the three subscale values, or simply sum all 9 items.

Each subscale contains 3 items, so the possible range of scores for each subscale is 3 through 15. The Total score has a range of 9 through 45.
